# Accuracies of genomic breeding values in American Angus beef cattle using K-means clustering for cross-validation

**DOI:** 10.1186/1297-9686-43-40

**Published:** 2011-11-28

**Authors:** Mahdi Saatchi, Mathew C McClure, Stephanie D McKay, Megan M Rolf, JaeWoo Kim, Jared E Decker, Tasia M Taxis, Richard H Chapple, Holly R Ramey, Sally L Northcutt, Stewart Bauck, Brent Woodward, Jack CM Dekkers, Rohan L Fernando, Robert D Schnabel, Dorian J Garrick, Jeremy F Taylor

**Affiliations:** 1 Department of Animal Science, Iowa State University, Ames, 50011, USA; 2 Division of Animal Sciences, University of Missouri, Columbia, 65211, USA; 3 Bovine Functional Genomics Laboratory, ARS, USDA, Beltsville, MD 20705, USA; 4 American Angus Association, 3201 Frederick Avenue, Saint Joseph, 64506, USA; 5 Igenity Livestock Business Unit, Merial Limited, Duluth, 30096, USA; 6 Institute of Veterinary, Animal and Biomedical Sciences, Massey University, Palmerston North, New Zealand

## Abstract

**Background:**

Genomic selection is a recently developed technology that is beginning to revolutionize animal breeding. The objective of this study was to estimate marker effects to derive prediction equations for direct genomic values for 16 routinely recorded traits of American Angus beef cattle and quantify corresponding accuracies of prediction.

**Methods:**

Deregressed estimated breeding values were used as observations in a weighted analysis to derive direct genomic values for 3570 sires genotyped using the Illumina BovineSNP50 BeadChip. These bulls were clustered into five groups using K-means clustering on pedigree estimates of additive genetic relationships between animals, with the aim of increasing within-group and decreasing between-group relationships. All five combinations of four groups were used for model training, with cross-validation performed in the group not used in training. Bivariate animal models were used for each trait to estimate the genetic correlation between deregressed estimated breeding values and direct genomic values.

**Results:**

Accuracies of direct genomic values ranged from 0.22 to 0.69 for the studied traits, with an average of 0.44. Predictions were more accurate when animals within the validation group were more closely related to animals in the training set. When training and validation sets were formed by random allocation, the accuracies of direct genomic values ranged from 0.38 to 0.85, with an average of 0.65, reflecting the greater relationship between animals in training and validation. The accuracies of direct genomic values obtained from training on older animals and validating in younger animals were intermediate to the accuracies obtained from K-means clustering and random clustering for most traits. The genetic correlation between deregressed estimated breeding values and direct genomic values ranged from 0.15 to 0.80 for the traits studied.

**Conclusions:**

These results suggest that genomic estimates of genetic merit can be produced in beef cattle at a young age but the recurrent inclusion of genotyped sires in retraining analyses will be necessary to routinely produce for the industry the direct genomic values with the highest accuracy.

## Background

Traditional methods of genetic evaluation depend on the accumulation and analysis of phenotypic and pedigree information to produce estimated breeding values (EBV). For a given selection intensity, response to selection measured in genetic standard deviations is proportional to the ratio of the accuracy of EBV and generation interval. In practice, accuracy increases but the generation interval is extended by waiting until the individual or offspring phenotypic records are available to estimate genetic merit, usually decreasing selection response. Genomic selection is a recently developed technology [1] that is beginning to revolutionize animal breeding. It is currently possible to genotype cattle for at least 50 000 single nucleotide polymorphisms (SNP) using a variety of assays, such as the BovineSNP50 [2], BovineHD (Illumina, San Diego, CA) or Axiom BOS 1 (Affymetrix, Santa Clara, CA) assays. These SNP panels can be used to produce direct genomic values (DGV), as proposed by Meuwissen et al. [1], via the estimation of marker effects from the analysis of a population with SNP genotypes and trait phenotypes (training set). The resulting estimates of SNP effects are then used in conjunction with SNP genotypes and trait phenotypes from a new group of animals (validation set) to evaluate the performance of the DGV prediction model. The accuracies of the resulting DGV, determined as the correlation between actual and predicted genetic merits, have only recently begun to be reported for traits in beef cattle [3-5], in contrast to numerous results from dairy cattle populations, including New Zealand Holstein-Friesian and Jerseys [6], North American Holstein [7], Australian Holstein-Friesian [8], Norwegian Red cattle [9] and Danish Holsteins [10].

Habier et al. [11] indicated that genomic selection uses genetic relationships among individuals and linkage disequilibrium (LD) between markers and quantitative trait loci (QTL) to improve the accuracy of DGV. The increase in accuracy of evaluation from using a genomic relationship matrix in traditional animal models comes from replacing an expected relationship matrix, which is conditional on the pedigree, with a realized matrix that is not influenced by missing pedigree information or violation of the assumption that the Mendelian sampling of parental gametes is drawn from a distribution with zero mean. In an earlier study, Nejati-Javaremi et al. [12] replaced the pedigree-based relationship matrix with a marker-based total allelic relationship matrix and documented its impact on reducing prediction error variance, hence, increasing the accuracy of evaluation. Saatchi et al. [13] and Habier et al. [14] have shown that the number of generations separating training and validation datasets influences accuracy, with lower accuracies occurring when this relationship is more distant.

The accuracy of DGV is key to the successful application of genomic selection in animal breeding but cannot be assessed in the training set. In practice, cross-validation can be performed in a sample of individuals that are related to those in the training set but that were not themselves included in training. The objective of this study was to investigate accuracies of DGV predicted for 16 economically important traits in US Angus beef cattle. We employed K-means clustering to pedigree estimates of the additive genetic relationships among the 3570 genotyped animals to partition animals into training and validation groups, with the aim of increasing within-group and decreasing between-group relationships for cross-validation. We also compared these results to those achieved from the more common practice of random allocation of individuals to the training and validation groups and from training on old animals and validating in young animals. In a national evaluation, the DGV could be considered as a correlated trait to that for which phenotypes are available for traditional estimation of EBV [15], in which case estimates of the genetic correlations between traits and respective DGV are required. We derived prediction equations for DGV and used these to estimate these correlations.

## Methods

### Genotype and phenotype data

A total of 3668 registered Angus bulls were genotyped with the BovineSNP50 BeadChip (Illumina, San Diego, CA) either at the University of Missouri (Columbia, MO) or GeneSeek (Lincoln, NE). Forty animals had genotypes that were inconsistent with the patrilineal pedigree and were removed, as well as 58 additional animals for which the genotype call rate (CR) was less than 95%, leaving 3570 Angus bulls born between 1955 and 2008 (Table 1). The DNA for each bull was obtained from cryopreserved semen provided by artificial insemination (AI) organizations, the National Animal Germplasm Program, the University of Maryland Wye herd, and numerous breeders of registered Angus cattle [16]. Scoring of marker genotypes was performed using Illumina's Beadstudio software (v3.2.23). Genotypes at a particular locus were filtered from further analysis according to the following criteria: (1) CR less than 90% (n = 472); (2) minor allele frequency (MAF) less than 1% (n = 5164); and (3) for autosomal and pseudoautosomal loci, Hardy-Weinberg equilibrium Chi-square statistic with 1 degree of freedom greater than 300 (n = 5745). These filters were not independent and resulted in 9360 of the 54 442 loci being removed, leaving 45 082 loci for analysis. Most animals had a few missing genotypes and these were imputed (0.45% of all genotypes) using fastPHASE [17].

**Table 1:** Birth year distribution of genotyped bulls (n = 3570)

Birth year	Number of bulls (n)
1955 to 1959	8
1960 to 1964	10
1965 to 1969	26
1970 to 1974	53
1975 to 1979	57
1980 to 1984	114
1985 to 1989	212
1990 to 1994	446
1995 to 1999	699
2000 to 2004	705
2005 to 2008	1240

Deregressed estimated breeding values (DEBV) were used as response variables to estimate SNP effects. An appropriate deregressing method, that removes parent average effects and which accounts for heterogeneous variance [18] was used to calculate DEBV from the EBV and their reliabilities of genotyped bulls and their sires and dams. Expected progeny differences (EPD) and their Beef Improvement Federation (BIF) accuracies for the genotyped bulls, their sires and dams were obtained from the American Angus Association (AAA) national cattle evaluation in August 2010. The EPD were transformed to EBV by multiplying by 2, and the corresponding reliabilities (R^2^) were obtained as:

$$R^{2}=1-{\left(1-BIF\text{\_}Accuracy\right)}^{2}.$$

In total, 16 traits were analyzed in this study: birth, weaning and yearling weights; yearling height; mature weight and height; maternal weaning weight (maternal milk); fat thickness; marbling score; rib eye muscle area; carcass weight; direct and maternal calving ease; scrotal circumference; docility and heifer pregnancy rate. The number of genotyped bulls with DEBV varied according to trait because some traits have only recently been introduced (e.g., heifer pregnancy) or because young bulls did not yet have progeny measurements on some traits (e.g., carcass composition traits). Heritabilities (reported by AAA, http://www.angus.org/Nce/Heritabilities.aspx), number of genotyped bulls with DEBV and their mean DEBV reliabilities for the studied traits are in Table 2.

**Table 2:** Heritability, number of genotyped bulls with DEBV and mean reliabilities of DEBV

Trait	**h**^**2**^	Number of bulls Reliability (R^2^)	
Birth weight	0.42	3203	0.79
Calving ease direct	0.18	3180	0.62
Calving ease maternal	0.12	1965	0.59
Carcass weight	0.40	2448	0.41
Docility	0.37	1363	0.50
Fat thickness	0.34	3155	0.40
Heifer pregnancy rate	0.13	698	0.48
Marbling	0.45	3199	0.44
Maternal weaning weight	0.14	2066	0.70
Mature weight	0.55	1320	0.64
Mature height	0.82	1290	0.64
Rib eye muscle area	0.51	3231	0.47
Scrotal circumference	0.43	2464	0.69
Weaning weight	0.20	3191	0.69
Yearling weight	0.45	2239	0.70
Yearling weight	0.49	2755	0.69

### Statistical model

In this study, all SNP markers that passed the filtering process were used as predictors with weighted DEBV used as response variables to estimate SNP effects. The Bayesian method presented in [19], which we will refer to as "BayesC," was used to estimate marker effects for genomic prediction. BayesC is related to both the BayesB and BLUP methods presented by Meuwissen et al. [1]. Like BLUP, BayesC assumes that SNP effects are drawn from a distribution with constant variance, but treats the common variance as unknown with a scaled inverse-chi square prior. Like BayesB, BayesC fits a mixture model that assumes some known fraction of markers (π) has zero effects. It has been shown that BayesC is less sensitive to prior assumptions than is BayesB [20].

For each trait the following model was fit to the DEBV data for training:

$$y_{i}=\mu +\overset{k}{\underset{j=1}{\sum }}z_{ij}u_{j}+e_{i}$$

where *y_i _*is the DEBV on animal i, *μ *is the population mean, k is the number of marker loci in the panel, *z_ij _*is allelic state (i.e., number of B alleles from the Illumina A/B calling system) at marker j in individual i, *u_j _*is the random effect for marker j, with $u_{j}\sim N\left(0,\sigma_{u}^{2}\right)$ (with probability 1 - π) or *u_j _*= 0 (with probability π), and *e_i _*is a residual with heterogeneous variance, depending on the reliability of the information on the bull [18]. Details concerning estimation of $\sigma_{u}^{2}$ are described in Kizilkaya et al. [19]. In this study, parameter π was assumed to be 0.995 for all analyses. Markov chain Monte Carlo (MCMC) methods with 41 000 iterations were used to provide posterior mean estimates of marker effects and variances after discarding the first 1000 samples that were used for burn-in. In preliminary analyses, all the genotyped bulls were included in the training set to obtain estimates of genetic and residual variances to construct the priors for the genetic and residual scale parameters.

The DGV for individual i within a validation set was derived as the sum of predicted effects of SNP posterior means over all k marker effects estimated in the training set:

$$DGV_{i}=\overset{k}{\underset{j=1}{\sum }}z_{ij}{û}_{j}$$

where DGV_i _is the DGV for individual i in the validation dataset, *z_ij _*is the marker genotype of individual i for marker j coded as for training, and ${û}_{j}$ is the estimated posterior mean effect of marker j over the 40 000 post burn-in samples. All analyses were performed using the GenSel software [21].

### Cross-validation

The accuracy of DGV was evaluated by pooling estimates using a 5-fold cross-validation strategy. Genotyped bulls were first divided into five mutually exclusive groups. In each training analysis, the data excluded one group to train on the remaining four groups to estimate marker effects, which were then used to predict DGV of individuals from the omitted group (validation set). This resulted in every bull having predicted DGV obtained without using its own DEBV, allowing that DEBV to be used in validation.

The K-means clustering method was applied to a dissimilarity or distance matrix containing elements of one minus the additive genetic relationship between pairs of animals to partition the genotyped bulls into five groups in which relatedness was increased within each group and decreased between each of the groups.

The dissimilarity matrix (D matrix) between genotyped individuals was computed from elements of the pedigree numerator relationship matrix (A matrix):

$$d_{ij}=1-\frac{a_{ij}}{\sqrt{a_{ii}\cdot a_{jj}}},$$

where *d_ij _*is a measure of pedigree distance between individual i and individual j, *a_ij _*is the additive genetic relationship between individual i and individual j, *a_ii _*(and *a_jj_*) are diagonal elements of the A matrix, which represent the relationship coefficient (including inbreeding) of individual i (or j) with itself. This formulation removes the effects of inbreeding and results in the diagonal elements of D being zero. We used the CFC Package [22] to construct the relationship matrix between the 3570 genotyped bulls, using pedigree information for all 109 594 known ancestors. Founder animals that appeared only once in the pedigree were pruned, which reduced the pedigree set to 91 001 animals. These individuals represented up to 64 pedigree generations. We used the Hartigan and Wong [23] algorithm, implemented using R [24] for K-means clustering. The maximum relationship coefficient (a_max_) was calculated between each animal and all other animals in each of the five partitioned groups, so that each animal had five a_max _values. The density distributions of the five a_max _values for all animals in a particular group were used to quantify the quality of the clustering. For comparative purposes, random clustering was also performed, with 5-fold cross-validation repeated for five replicates for each of the studied traits.

### Validation on younger animals

In practical livestock applications, training will occur on historical animals, and the target population for implementation of genomic selection may include but not be limited to their offspring. Thus, it has been common to validate DGV on progeny (selection candidates) or in young animals, as in US dairy cattle [7], where essentially all historic and currently active AI sires have been genotyped and are used in training. In contrast, many beef cattle sires are used only by natural mating, and cross-validation using less related training sets, rather than immediate progeny, may more appropriately reflect the DGV accuracies achieved in practice. However, for comparison to the dairy results, we also partitioned the genotyped bulls into two groups according to their birth year and trained on the older bulls and validated in the younger animals. Different birth years were used as thresholds for this partitioning for each trait so that about one-fifth of the individuals were always in the validation set. The numbers of individuals in the training and validation sets and the birth year range for individuals in the validation set for each trait are in Table 3.

**Table 3:** Numbers of individuals and birth-year range in the training and validation sets

	Training		Validation	
Trait	Number	Birth year	Number	Birth year
Birth weight	2515	1957-2006	688	2007-2008
Calving ease direct	2507	1957-2006	673	2007-2008
Calving ease maternal	1600	1957-2001	365	2002-2008
Carcass weight	1906	1957-2004	542	2005-2008
Docility	1130	1963-2004	233	2005-2008
Fat thickness	2268	1957-2006	887	2007-2008
Heifer pregnancy rate	565	1968-2001	133	2002-2008
Marbling	2308	1957-2006	891	2007-2008
Maternal weaning weight	1671	1957-2001	395	2002-2008
Mature height	1066	1957-2000	224	2001-2008
Mature weight	1072	1957-2000	248	2001-2008
Rib eye muscle area	2339	1957-2006	892	2007-2008
Scrotal circumference	2025	1957-2004	439	2005-2008
Weaning weight	2510	1957-2006	681	2007-2008
Yearling height	1843	1957-2003	396	2004-2008
Yearling weight	2182	1957-2004	573	2005-2008

Numbers and ranges from partitioning of older animals for training and younger animals for validation

### Accuracy of DGV

The accuracy of DGV could be defined as the correlation between true genetic values and $\text{DGV}\left({\hat{\rho }}_{gĝ}\right)$, which could be computed as:

$${\hat{\rho }}_{gĝ}=\frac{{\hat{\sigma }}_{g,DGV}}{\sigma_{g}^{2}{\hat{\sigma }}_{DGV}^{2}},$$

where ${\hat{\rho }}_{gĝ}$ is the accuracy of DGV, ${\hat{\sigma }}_{g,DGV}$ is the covariance between true genetic values and DGV, $\sigma_{g}^{2}$ and ${\hat{\sigma }}_{DGV}^{2}$ are the population additive genetic and the sample DGV variances, respectively. The true genetic values of genotyped animals are not available but the DEBV could be used here with the same expectation of covariance since $E\left[{\hat{\sigma }}_{DEBV,DGV}\right]=E\left[{\hat{\sigma }}_{\left(g+e,DGV\right)}\right]={\hat{\sigma }}_{g,DGV}+{\hat{\sigma }}_{e,DGV}={\hat{\sigma }}_{g,DGV}$ if we assume, ${\hat{\sigma }}_{e,DGV}=0$, where ${\hat{\sigma }}_{DEBV,DGV}$ is the covariance between DEBV and DGV. The following formula was used to measure the accuracy of DGV:

$${\hat{\rho }}_{gĝ}=\frac{{\hat{\sigma }}_{DEBV,DGV}}{\sqrt{\sigma_{g}^{2}{\hat{\sigma }}_{DGV}^{2}}}$$

This formula is a generalization of the approach that would be used when validation occurs in a set of individuals with phenotypes and the correlation between DGV and phenotypes is standardized by dividing by the square root of the heritability. The additive genetic variance $\left(\sigma_{g}^{2}\right)$ was derived from:

$$\sigma_{g}^{2}=h^{2}\sigma_{p}^{2},$$

where *h*^2 ^is the trait heritability as reported by AAA (Table 2) and $\sigma_{p}^{2}$ is the phenotypic DEBV variance estimated from the primary analysis using all genotyped animals in the training set (as the sum of the estimated genetic and residual variances).

The genotyped bulls represent birth years from 1955-2008, a period with considerable genetic trend for some traits. We estimated the generation interval in the pedigree of the genotyped Angus cattle to be 4.99 years (data not shown), which is the average age of bulls within the pedigree born between 1941 and 1990 (the part of the pedigree that captured most animals) at the birth of their progeny. Accordingly, we fitted contemporary groups defined by year of birth (in 5-year intervals) as fixed effects to remove any effects of selection that could inflate correlations. The sample covariance and sample variances from each 5-year interval were pooled according to their respective degrees of freedom.

### Regression of DEBV on DGV

The extent of prediction bias can be judged by comparing the regression of true breeding value (here, DEBV) on predicted breeding value (DGV), with its expected value of 1 for each trait. Hence, the regression coefficients were calculated for each trait using simple linear regression of DEBV on DGV.

### Parent average and genomic-enhanced breeding values

Conventional genetic evaluation systems generate parent average (PA) predictions that can be used to facilitate animal selection prior to the measurement of individual or offspring phenotypes. Thus, PA provides an accuracy benchmark for comparison to the accuracy of DGV. However, the available PA information in our dataset does not represent that available on the parents of the genotyped bulls at the time of their birth. Hence, we tried to exclude information of the genotyped bull from the cumulative information available on his parents using the Garrick et al. [18] deregression method to create an adjusted PA (PA_adj_) for each genotyped bull. Also, genomic-enhanced breeding values (GEBV) blending PA_adj _and DGV obtained from K-means clustering and cross-validation, was constructed as:

$$\text{GEBV}={\text{b}}_{1}PA_{adj}+{\text{b}}_{2}\text{DGV},$$

where b_1 _and b_2 _were estimated using multiple regression with DEBV as the response variable. The accuracies of PA_adj _and GEBV were calculated with the same formula as for DGV for each trait. Contemporary groups within each of the five partitioned groups based on 5-year birth intervals were considered as fixed effects to allow for the effects of genetic trends in each trait and fair comparisons with the accuracies of DGV.

### Genetic correlations between traits and DGV

For each of the 16 traits, we applied a weighted bivariate analysis using DGV of genotyped animals from the five validation sets obtained by K-means clustering and 5-fold cross-validation and their DEBV to estimate variance and covariance components. The model was:

$$\left[\begin{array}{c} DEBV\\ DGV \end{array}\right]=\left[\begin{array}{cc} X_{1} & 0\\ 0 & X_{2} \end{array}\right]\left[\begin{array}{c} \beta_{1}\\ \beta_{2} \end{array}\right]+\left[\begin{array}{cc} Z_{1} & 0\\ 0 & Z_{2} \end{array}\right]\left[\begin{array}{c} \alpha_{1}\\ \alpha_{2} \end{array}\right]+\left[\begin{array}{c} e_{1}\\ e_{2} \end{array}\right]$$

where *β*_1 _and *β*_2_, are vectors of fixed effects (only the trait mean for *β*_1 _but class effects of the five K-means partitioned groups for *β*_2_); *α*_1 _and *α*_2_, are vectors of random additive genetic effects for the two traits, $Var\left(\alpha_{1}\right)=A\sigma_{\alpha_{1}}^{2}$, $Var\left(\alpha_{2}\right)=A\sigma_{\alpha_{2}}^{2}$ and $Cov\left(\alpha_{1},\alpha_{2}\right)=A\sigma_{\alpha_{1},\alpha_{2}}$, where A is the pedigree numerator relationship matrix; e_1 _and e_2_, are vectors of mutually uncorrelated random residual effects for the two traits, $Var\left(e_{1}\right)=I\sigma_{e_{1}}^{2}$ and $Var\left(e_{2}\right)=W\sigma_{e_{2}}^{2}$, where I is an identity matrix and W is a diagonal matrix containing the r-inverse weights according to the reliability of the bulls' DEBV [18], which are the same weights as used in the estimation of SNP effects; X and Z are known design matrices for fixed effects and random additive genetic effects, respectively. The purpose of fitting this model was to estimate the genetic correlation between the DGV and the trait (*r*_*g*(*DGV,T*)_), which is required to pool DGV and traditional EBV in national genetic evaluation [15], the square of which represents the proportion of genetic variance accounted for by the genomic information if the DGV has a heritability of 1. Variance components were estimated by restricted maximum likelihood (REML) using the ASReml v3.0 software package [25].

## Results

### K-means and random clustering

**Figure 1:**
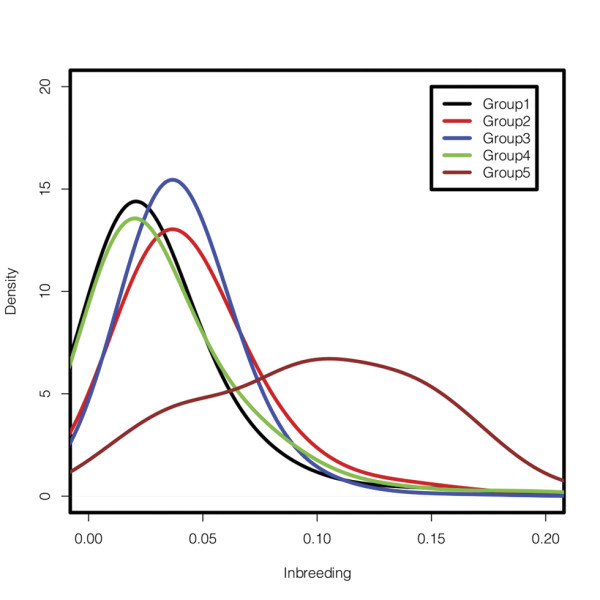
**The density distribution of inbreeding coefficients in each clustered group**.

Table 4 shows the number of individuals, the average inbreeding coefficients, and a_max _within and between the K-means clustered groups. Figure 1 shows the density distribution of inbreeding coefficients for each of the five groups. Group 5, with 220 bulls, was characterized with high levels of inbreeding (about 10%) and represents animals from the Wye Angus herd formed from an importation of bulls from the British Isles and then closed to new germplasm in 1958 (http://wyeangus.umd.edu/History.cfm), along with two bulls from Dunlouise Angus (http://www.dunlouiseangus.com/), a Scottish herd of native origin Angus. Some bulls produced within the Wye herd have been used in artificial insemination within the national Angus herd, leading to nontrivial relationships of group 5 with the other groups.

**Table 4:** The number of individuals and the averages (± standard deviation)

Groups	1	2	3	4	5
Number	1033	885	1084	348	220
Birth year	1995.2 ± 10.3	1999.3 ± 6.2	2003.9 ± 4.6	1999.5 ± 5.4	1985.2 ± 10.9
Inbreeding	0.033 ± 0.036	0.047 ± 0.033	0.042 ± 0.026	0.035 ± 0.034	0.102 ± 0.054
a_ij _within group	0.038 ± 0.036	0.099 ± 0.060	0.088 ± 0.057	0.161 ± 0.086	0.188 ± 0.100
a_max _within group	0.42 ± 0.14	0.49 ± 0.10	0.45 ± 0.12	0.49 ± 0.11	0.58 ± 0.09
a_max _between groups	0.18 ± 0.14	0.23 ± 0.17	0.23 ± 0.18	0.23 ± 0.18	0.11 ± 0.15

For birth year, inbreeding coefficients, within group pedigree relationships (a_ij_), maximum within and between group relationships (a_max_) for five partitioned groups after K-means clustering

**Figure 2:**
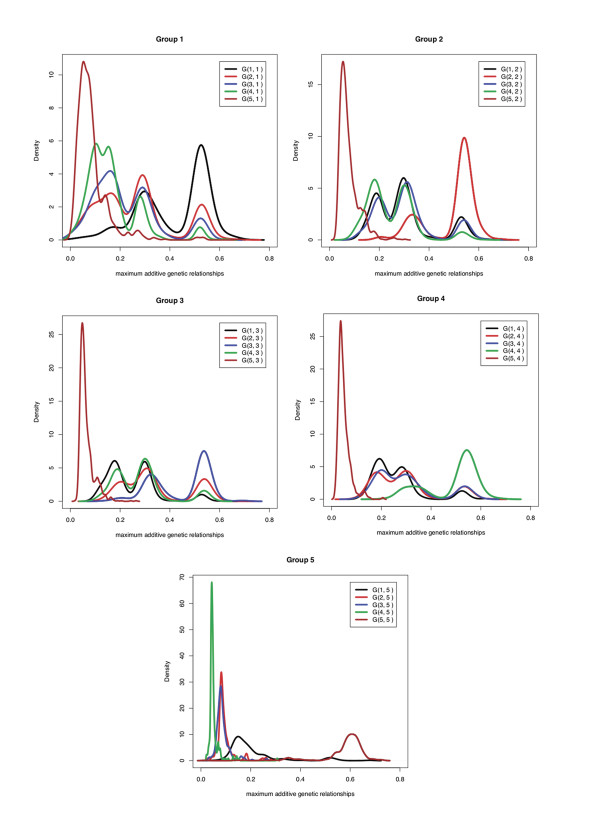
**The density distribution of the maximum additive genetic relationships (a_max_)**. The density distribution of the maximum additive genetic relationships (a_max_) between each individual in a particular group and all animals in the same or different groups formed by K-means clustering

Table 4 shows that a_max _values within a group are much larger than the average of the a_max _values of a group with the other four groups. The greatest difference between these a_max _values was for group 5, which had the highest within group a_max _(0.58) and the lowest a_max _with the other groups (0.11). These values indicate that, on average, any animal in group 5 had some relative within the group with an additive relationship exceeding 0.5, the relationship of a non-inbred parent with offspring or with non-inbred full-sibs. In contrast, on average, any animal in group 5 had an additive relationship of less than 0.125 with its closest relative in the four other groups, less than the relationship of a non-inbred individual with its great-grandparent or between cousins. Figure 2 expands on the information in Table 4 by showing the density distribution of a_max _of each individual in a particular group with all animals in the same or different groups. The a_max _statistic has high densities within each group around 0.5, representing sire-son or full-sib relationships, and around 0.25, representing grandparent-offspring or half-sib relationships, but a low density in the equivalent regions between groups. Figure 2 shows that the K-means clustering partitioned individuals into related groups with decreased relationship between groups. These results also show that group 5 is a distinct group that is less related to the other groups.

**Figure 3:**
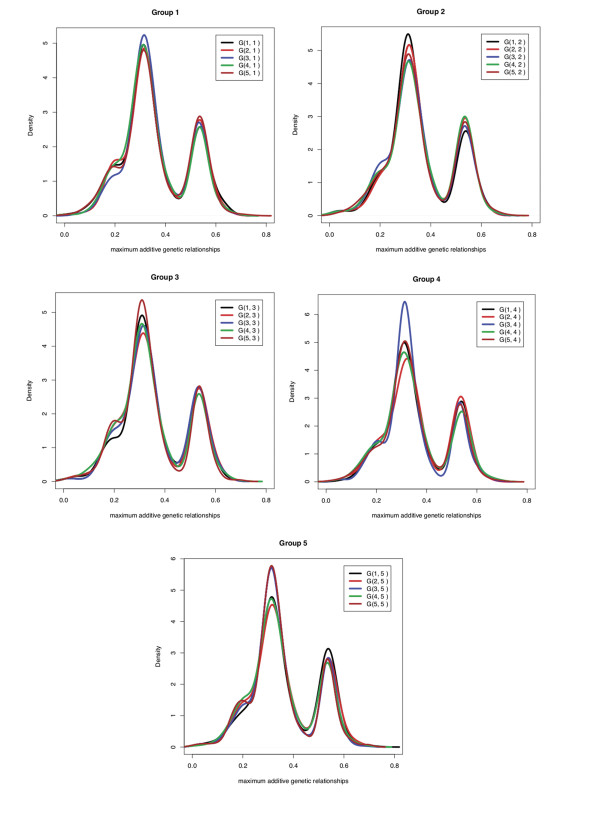
**The density distribution of the maximum additive genetic relationships (a_max_)**. The density distribution of the maximum additive genetic relationships (a_max_) between each individual in a particular group and all animals in the same or different groups when groups were formed at random

The results from random clustering were markedly different from those from K-means clustering. With random clustering, there were no significant differences between the average within and between group a_max _values, which ranged from 0.34 to 0.37 over the five randomly clustered replicates. Figure 3 shows the a_max _density distribution for each individual in a particular group with all animals in the same or different groups for one of the randomly clustered groups. There were no significant differences in relationships between the groups. The a_max _statistic has high densities around 0.25 and 0.5, both within and between groups, which indicates that most individuals within each group have at least one close relative in each of the other groups.

### Accuracy of DGV with K-means and random clustering

The accuracies of DGV based on training in four groups partitioned by K-means clustering and predicting the other group for birth, weaning and yearling weights and averaged over all 16 traits are in Table 5. The results show considerable variation in accuracy between groups. Accuracies were lowest for group 5, which was less related to the other groups. Table 6 presents the pooled accuracy of DGV by K-means clustering and 5-fold cross-validation for all studied traits. Also, phenotypic and genetic variances (?20? and ?12?, respectively) used for standardization of the covariance and the regression of DEBV on DGV for each trait is shown in Table 6.

**Table 5:** Accuracies of DGV for five K-means clustered groups and the pooled accuracy

Trait	Birth weight	Weaning weight	Yearling weight	All traits
Group 1	0.561	0.422	0.434	0.450
Group 2	0.703	0.323	0.335	0.463
Group 3	0.464	0.320	0.360	0.446
Group 4	0.542	0.235	0.260	0.377
Group 5	0.356	0.184	0.273	0.258
Pooled	0.554	0.333	0.356	0.441

For birth, weaning and yearling weight; and averaged across all 16 studied traits

**Table 6:** Phenotypic and additive genetic variance; accuracies of DGV and regressions of DEBV on DGV

			K-means		Random	
Trait	$\sigma_{p}^{2}$	$\sigma_{g}^{2}$	${\hat{\rho }}_{g\hat{g}}$	${\hat{\beta }}_{y,x}$	${\hat{\rho }}_{g\hat{g}}$	${\hat{\beta }}_{y,x}$
Birth weight (kg)	20.63	8.67	0.554	0.879	0.700	0.953
Calving ease direct (%)	825.05	148.51	0.488	0.942	0.617	1.007
Calving ease maternal (%)	1313.37	157.6	0.416	1.181	0.571	1.277
Carcass weight (kg)	1535.06	614.03	0.471	1.130	0.689	1.208
Docility (%)	1634.60	604.8	0.218	0.614	0.490	1.150
Fat thickness (mm)	4.8	1.63	0.603	1.113	0.793	1.211
Heifer pregnancy rate (%)	1031.74	134.13	0.269	1.337	0.378	1.580
Marbling (units)	0.797	0.359	0.690	1.058	0.817	1.041
Maternal weaning weight (kg)	1160.85	162.52	0.318	0.702	0.492	0.829
Mature height (mm)	494.03	405.13	0.359	0.977	0.819	1.091
Mature weight (kg)	4638.66	2551.28	0.312	0.898	0.769	1.125
Rib eye muscle area (mm^2^)	430.32	219.35	0.601	0.993	0.694	0.958
Scrotal circumference (mm)	839.98	361.19	0.487	0.916	0.600	0.983
Weaning weight (kg)	1558.04	311.61	0.333	0.597	0.534	0.760
Yearling height (mm)	344.68	155.19	0.575	1.015	0.850	1.011
Yearling weight (kg)	2049.19	1004.12	0.356	0.642	0.573	0.790

Phenotypic variance $\left(\sigma_{p}^{2}\right)$ and additive genetic variance $\left(\sigma_{g}^{2}\right)$ obtained from the primary analysis using all genotyped animals in the training set; the accuracy of DGV $\left({\hat{\rho }}_{gĝ}\right)$ and the regression of DEBV on DGV $\left({\hat{\beta }}_{y,x}\right)$ obtained by K-means and random clustering (five replicates) along with 5-fold cross-validation for 16 traits

Accuracies of DGV varied and ranged from 0.22 to 0.69, with an average of 0.44 over all traits. Among the post-natal growth traits, the accuracies of DGV for birth weight and yearling height were higher than for weaning and yearling weight. Accuracies of DGV for carcass traits were generally higher than for growth traits. Marbling and rib eye muscle area had the highest DGV accuracies among all the studied traits. Accuracies of DGV for reproductive and behavioral traits were considerably lower than for other traits. Docility and heifer pregnancy rate had the lowest DGV accuracies (less than 0.3) among all studied traits.

Training was generally less accurate for traits with fewer animals with DEBV (Table 2 and Table 6). Traits exhibiting the highest bias, having regressions of DEBV on DGV departing from 1, also exhibited less accuracy, regardless of the number of animals with DEBV. For example, rib eye muscle area and yearling height, which had the highest accuracy, exhibited little bias (deviations from 1 of 0.007 and 0.015, respectively), while weaning weight and docility, which had low accuracies, had the most bias (0.403 and 0.386, respectively). In general, predictions tended to be biased downwards, as the average regression coefficient was 0.937 across all traits.

Table 6 also presents the average regression coefficients and the pooled accuracy of DGV obtained by random clustering and 5-fold cross-validation in five replicates for all traits. The accuracies of these DGV were considerably higher for all traits than the corresponding accuracies obtained by K-means clustering. The average of DGV accuracies over all traits was 0.65, which is 0.21 higher than the average of DGV accuracies obtained by K-means clustering.

### Accuracy of DGV with validation in young animals

**Figure 4:**
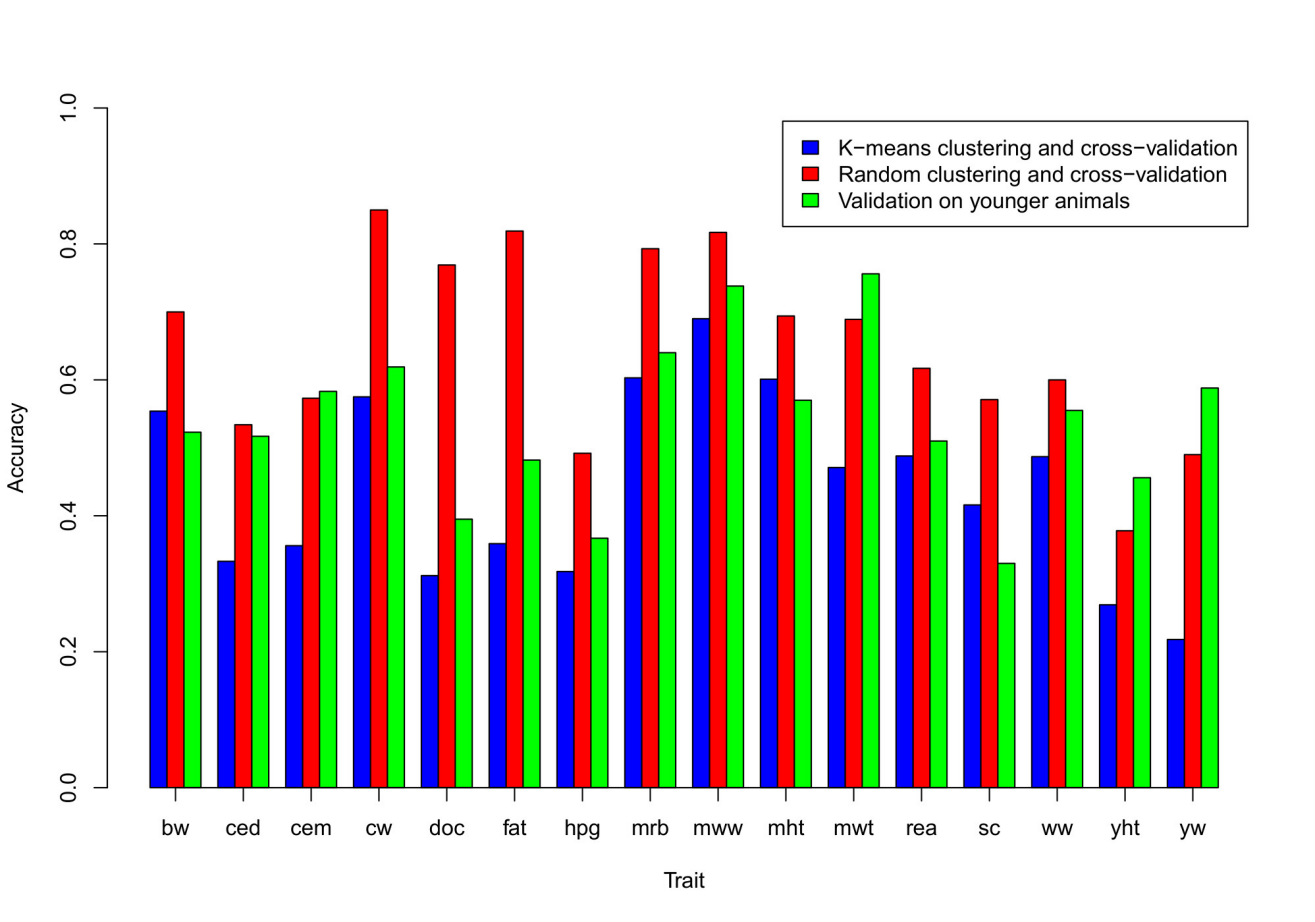
**The accuracies of DGV obtained by three different methods**. Comparison of the accuracies of DGV obtained by three different methods: K-means clustering and cross-validation, random clustering and cross-validation and validation on younger animals); traits- bw: birth weight, ced: calving ease direct, cem: calving ease maternal, cw: carcass weight, doc: docility, fat: fat thickness, hpg: heifer pregnancy rate, mrb: marbling, mww: maternal weaning weight, mwt: mature weight, mht: mature height, rea: rib eye area, sc: scrotal circumference, ww: weaning weight, yht: yearling height and yw: yearling weight

The comparison of accuracies of DGV obtained by K-means or random clustering and cross-validation methods with the accuracies of DGV obtained from training on older animals and validating in younger animals is shown in Figure 4. The accuracies of DGV obtained from training on older animals and validating in younger animals were higher than the accuracies obtained from K-means clustering and cross-validation for all traits except calving ease maternal. However, these accuracies were lower than the accuracies of DGV obtained by random clustering and cross-validation for most traits.

### Parent average and GEBV

**Figure 5:**
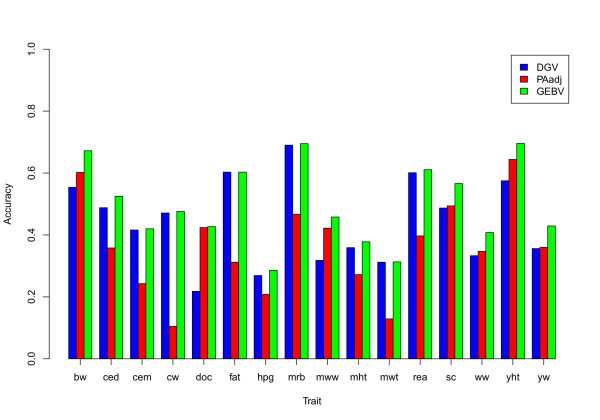
**The accuracies of DGV (from K-means clustering), PA_adj _and GEBV**. Comparison between the accuracies of DGV, adjusted parent average (PA_adj_) and GEBV from combining PA_adj _and DGV for all studied traits; traits: bw: birth weight, ced: calving ease direct, cem: calving ease maternal, cw: carcass weight, doc: docility, fat: fat thickness, hpg: heifer pregnancy rate, mrb: marbling, mww: maternal weaning weight, mwt: mature weight, mht: mature height, rea: rib eye area, sc: scrotal circumference, ww: weaning weight, yht: yearling height and yw: yearling weight

Figure 5 shows the accuracy of PA_adj _and the GEBV (blending PA_adj _and DGV) versus the accuracy of DGV for all studied traits. In general, the genomic prediction accuracies were similar to the PA_adj _accuracies for most traits. However, for all growth traits except mature weight and mature height, the accuracy of PA_adj _was slightly higher than the accuracy of DGV, while the accuracy of DGV exceeded that of PA_adj _for all carcass and some reproductive traits (calving ease direct and maternal). The accuracy of PA_adj _was higher than the accuracy of DGV for docility. The accuracy of the GEBV obtained from blending PA_adj _and DGV information did not substantially improve the genetic prediction over the more accurate source of information (PA_adj _or DGV) for any trait. Regression coefficients and the correlation between PA_adj _and DGV are shown in Table 7. As expected, regression coefficients of DEBV on DGV were greater than the regressions of DEBV on PA_adj _for traits for which the DGV was more accurate than PA_adj _and *vice versa*. The regression coefficient for DGV ranged from 0.17 (for docility) to 1.20 (for carcass weight), while regression coefficients for PA_adj _ranged from -0.19 (for carcass weight) to 0.82 (for docility). The correlation between DGV and PA_adj _ranged from 0.27 to 0.65, with an average of 0.48 across traits.

**Table 7:** Regression coefficients of PA_adj _and DGV on DEBV (b_1 _and b_2_, respectively); the correlation between PA_adj _and DGV (Cor(PA_adj_, DGV))

Trait	b1	b2	Cor(PA_adj_, DGV)
Birth weight	0.66	0.54	0.41
Calving ease direct	0.38	0.80	0.51
Calving ease maternal	0.13	1.09	0.45
Carcass weight	-0.19	1.20	0.51
Docility	0.82	0.17	0.27
Fat thickness	0.03	1.10	0.54
Heifer pregnancy rate	0.23	1.09	0.40
Marbling	0.17	0.97	0.65
Maternal weaning weight	0.69	0.42	0.60
Mature height	0.21	0.81	0.48
Mature weight	0.04	0.87	0.37
Rib eye muscle area	0.24	0.89	0.61
Scrotal circumference	0.49	0.60	0.54
Weaning weight	0.50	0.42	0.38
Yearling height	0.70	0.56	0.47
Yearling weight	0.54	0.46	0.46

### Genetic correlations between traits and DGV

Table 8 presents the estimated heritabilities of DGV and traits and the genetic correlations between the trait and their respective DGV. The estimated variance and covariance for heifer pregnancy rate had high standard errors due to an inadequate number of training bulls and are not presented. Heritabilities of DGV for the remaining traits were more than 0.80 and the genetic correlations between trait and DGV ranged from 0.15 (for docility) to 0.80 (for carcass weight). Heritabilities of DGV less than one reflect genotyping errors (<0.5%), effects of missing pedigree information and within-family selection on the estimation of the pedigree relationship matrix, and differences in the prediction equation between validation groups. Estimates of genetic correlations between the trait and DGV reflect the accuracies of DGV pooled across groups. The estimated heritabilities for traits using the bivariate animal model were lower than the corresponding heritabilities reported by AAA (Table 2).

**Table 8:** Estimates of heritability and genetic correlations between traits and their respective DGV

Trait	$h_{DGV}^{2}$	$h_{T}^{2}$	*r*_*g*(*DGV*,*T*)_
Birth weight	0.87 ± 0.03	0.37 ± 0.03	0.58 ± 0.03
Calving ease direct	0.83 ± 0.03	0.11 ± 0.01	0.64 ± 0.03
Calving ease maternal	0.95 ± 0.02	0.03 ± 0.01	0.67 ± 0.06
Carcass weight	0.84 ± 0.03	0.16 ± 0.03	0.80 ± 0.06
Docility	0.75 ± 0.04	0.34 ± 0.04	0.15 ± 0.06
Fat thickness	0.85 ± 0.03	0.20 ± 0.02	0.68 ± 0.05
Marbling	0.86 ± 0.02	0.32 ± 0.02	0.73 ± 0.05
Maternal weaning weight	0.86 ± 0.03	0.09 ± 0.01	0.41 ± 0.04
Mature height	0.89 ± 0.04	0.69 ± 0.04	0.34 ± 0.06
Mature weight	0.84 ± 0.04	0.34 ± 0.04	0.41 ± 0.06
Rib eye muscle area	0.90 ± 0.02	0.41 ± 0.03	0.73 ± 0.04
Scrotal circumference	0.82 ± 0.03	0.24 ± 0.03	0.68 ± 0.04
Weaning weight	0.81 ± 0.03	0.14 ± 0.01	0.49 ± 0.03
Yearling height	0.93 ± 0.02	0.40 ± 0.03	0.45 ± 0.04
Yearling weight	0.84 ± 0.03	0.39 ± 0.03	0.56 ± 0.03

Heritability of $\text{DGV}\left(h_{DGV}^{2}\pm \text{SE}\right)$, heritability of traits $\left(h_{T}^{2}\pm \text{SE}\right)$ and genetic correlations between traits and their respective DGV(*r*_*g*(*DGV*,*T*) _± SE) estimated from bivariate animal models

## Discussion

The accuracy of DGV is critical to determine the utility of DGV in relation to genotyping costs. In simulation studies, the correlation between DGV and true breeding values (TBV) has been used to represent the accuracy of DGV. However, in field data, TBV are not available and the correlation between DGV and the response variable (phenotype records, EBV, DEBV, etc.) typically underestimate the accuracy of DGV due to the contribution of environmental effects and random error to the response variable. Habier et al. [14] estimated marker effects using daughter yield deviations (DYD) of dairy bulls and divided the correlation between DGV and DYD by the average accuracy of the DYD to estimate the correlation between DGV and TBV. Su et al. [10] used the average accuracy of EBV to adjust the simple correlation between DGV and EBV (the response variable). VanRaden et al. [7] divided the GEBV accuracy by the mean accuracy of the DYD and then added the difference between the published and observed accuracy of PA to calculate the realized genomic accuracy. However, using the mean accuracy as an adjustment factor does not consider the heterogeneous error variance, which is associated with the DEBV of different bulls and this may lead to a bias. In this study, accuracy was obtained by standardizing the estimated covariance between DEBV and DGV using the genetic variance.

Reports on the accuracy of DGV for beef cattle are scarce. Rolf et al. [26] found low accuracies of about 0.3 for average daily feed intake, residual feed intake and average daily gain, when a genomic relationship matrix was used for 2405 genotyped Angus steers and sires. In dairy cattle, Harris et al. [6] reported accuracies of DGV for young bulls with no daughter information ranging from 0.71 to 0.82 for milk production traits, live weight, fertility, somatic cell count and longevity, compared to an average accuracy of 0.58 for PA in a New Zealand Holstein population. In their study, accuracies of DGV for linear type traits were lower than for production traits and ranged from 0.63 to 0.71, compared to an average of 0.56 for PA for these traits. The average accuracy from combining DGV and PA for 27 traits in the North American Holstein population reported by VanRaden et al. [7] was 0.71, compared to 0.52 from PA alone. Accuracies for GEBV combining DGV and national EBV for 12 Dutch Holstein traits ranged from 0.52 to 0.82, with an average of 0.71 [27]. Luan et al. [9] reported accuracies of DGV for milk, fat and protein yields, first lactation mastitis and calving ease ranging from 0.12 to 0.62 using a small sample (500 genotyped bulls) of Norwegian Red cattle. Su et al. [10] reported simple correlations between DGV and published EBV (as a response variable) ranging from 0.50 to 0.84, with an average of 0.65 and adjusted correlations ranging from 0.70 to 0.85, with an average of 0.74 for 18 traits in a Danish Holstein population. These authors also reported that simple and adjusted accuracies were 0.36 and 0.51 higher than the accuracies of PA. Hayes et al. [8] reported accuracies of DGV ranging from 0.37 to 0.74 for five simple and index traits in Australian Holstein cattle. In general, however, it is difficult to compare the accuracies from different studies because of differences in trait heritabilities, data types (phenotypes, EPD, DYD or DEBV), training and validation set sizes, validation methods (set definition) and statistical methods to estimate marker effects.

In general, the DGV accuracies obtained here by K-means clustering and 5-fold cross-validation were lower than reported for dairy cattle for traits with similar heritabilities. For example, Su et al. [10] used 5-fold cross-validation in a genotyped group of 3330 bulls (almost the same size as this study) and reported modified accuracies of 0.71 and 0.72 for birth index and calving index traits. Accuracies obtained for similar traits (birth weight and calving ease direct) in our study were 0.55 and 0.49, respectively. The main reason for the lower accuracies observed in our study is the validation method, where we deliberately tried to minimize the relationship between members of the training and validation sets by K-means clustering. Habier et al. [11] showed that DGV use realized genetic relationships among individuals to increase the accuracy of DGV (i.e., the accuracy of a DGV on a selection candidate decreases as the average genetic relationship to the training set individuals decreases). Thus, the accuracies of DGV obtained by random clustering or from training in older animals and prediction in younger animals (which can generate larger genetic relationships between members of the training and validation sets) are higher than accuracies of DGV obtained by K-means clustering.

Another reason for the lower accuracies obtained in our study is that the accuracy of genotyped bulls EBV (used to derive the DEBV response variable) is lower in beef than in dairy cattle because artificial insemination is less used [28]. The average accuracy of EBV for the genotyped bulls across traits was only 0.77 in this study but 0.89 in the study by Su et al. [10]. The accuracy of DGV will increase as the accuracy of EBV increases because the response variable will be closer to the true breeding value. Another reason for the lower accuracies in comparison to those from dairy cattle studies could be the different extents and patterns of LD, which exist among breeds due to differing population histories and effective population sizes (N_e_). De Roos et al. [29] found that, for distances between 100 kb (kilobase) and 1 Mb (Megabase), Dutch Holstein-Friesian (HF) had the highest LD, followed by Dutch Red and White HF, then Australian Angus and New Zealand Jersey, and finally Australian HF and New Zealand HF, demonstrating that the extent of LD differs between subpopulations within a breed such as HF. The subpopulations have different historical backgrounds and effective population sizes. Prasad et al. [30] showed that there are regions of high and low LD across the chromosomes in both the Angus and Holstein breeds and a clear difference was observed in the pattern of LD between the two breeds. A difference in the extent of LD over different chromosomes has also been reported by McKay et al. [31] in Angus and other breeds.

Another reason for the lower accuracies of DGV observed in this study could be due to different N_e _between breeds. Goddard and Hayes [32] showed that more animals are needed for training to obtain the same accuracy with increasing effective population size. De Roos et al. [29] estimated an effective population size of about 100 for Dutch black-and-white Holstein-Friesian bulls, Dutch red-and-white Holstein-Friesian bulls, Australian Holstein-Friesian bulls, Australian Angus animals, New Zealand Friesian cows, and New Zealand Jersey cows. An effective population size less than 100 was estimated for the North American Holstein population by Kim and Kirkpatrick [33]; N_e _= 103 for German Holstein cattle by Qanbari et al. [34]; and N_e _= 49, 53 and 47 for Danish Holstein, Danish Jersey and Danish Red cattle by Sorensen et al. [35]. Marquez et al. [36] reported a high effective population size (N_e _= 445) for American Red Angus beef cattle, whereas a relatively low effective population size (N_e _= 85) was estimated for American Hereford beef cattle by Cleveland et al. [37]. We estimated a high effective population size N_e _= 654 ± 31 for American Angus beef cattle (data not shown), which is much higher than that found for North American Holstein and American Hereford beef cattle.

DGV were generally less accurate for traits that had fewer animals with DEBV. The importance of training population size on the accuracies of DGV has been shown in several studies [1,38]. Although training population size and the accuracy of DEBV have a large effect on the accuracy of DGV, the accuracy also depends on other factors such as the genetic architecture of the trait (assumptions about *π*) and the LD between markers and with genes that affect the trait, which could differ between traits. Hayes et al. [39] showed that the accuracy of genomic predictions is higher for traits with some loci having large effects than for traits with no loci of large effect. The difference in the accuracy of DGV between low and high heritability traits was relatively small. In most studies using simulated data, the phenotype of genotyped individuals is used to estimate marker effects and in this case heritability has been shown to affect the accuracy of genomic prediction [38,40]. In this study, we used DEBV to estimate marker effects and DGV. Using DEBV as the response variable is expected to make the DGV accuracy less dependent on heritability and more a function of the EBV accuracy. Here, EBV were predicted from a fairly large dataset, resulting in relatively high accuracies even for traits with a low heritability. Low heritability traits such as fitness traits have been largely ignored in livestock breeding due both to their low heritability and difficulty in recording. However, bulls can have a high accuracy for a low heritability trait if they have sufficient progeny. Thus, these traits could be included in genomic selection programs if suitable training sets could be formed.

Comparing the DGV accuracies obtained from K-means clustering and cross-validation to those for PA_adj _indicated that the accuracies were similar for most traits. The superiority of DGV accuracies over PA_adj _accuracies for carcass traits could be due to the lower accuracy of parental EBV for these traits, which are measured in limited numbers of progeny of these parents at slaughter. The PA_adj _accuracies obtained in this study were higher than those reported in other studies [7,8] primarily because the available PA information in our dataset does not represent that available on the parents of the genotyped bulls at the time of their birth. The deregression method used here only excluded information for the genotyped bull from the cumulative information available on his parents and did not exclude information from other relatives, including grand-progeny, which are informative for the meioses that produced the bull being deregressed and the majority of the genotyped bulls belonged to large patrilineages. VanRaden et al. [7] showed that combined predictions (PA and genomic predictions) were more accurate than PA (0.22 to 0.62 greater with nonlinear genomic predictions) in North American Holstein bulls. In this study, the accuracy of GEBV obtained by combining DGV and PA_adj _information did not increase the accuracy for most traits, suggesting that the PA_adj _may not be fully independent of the Mendelian sampling effect that produced the bull for which deregression was performed. The gain from combining DGV with PA_adj _depends on the accuracy of DGV and PA_adj _and the correlation between them. Less gain in accuracy is expected from combined values if the two information sources are highly correlated. In this study, the accuracies of PA were higher than those available at the time of an animal's birth because the older animals in this population were all ancestors of the younger animals. Thus, in practice, the accuracies of PA on young selection candidates would be lower than found here because the PA would not contain information on grand-progeny and more gain could be expected from combining DGV with PA information. In addition, if the animal's own record is available before the selection decision, we have the advantage of that record in addition to PA. In this situation, less gain could be expected from combining DGV with an animal model EBV that included the individual record. However, in beef cattle, the only observation we typically have on a young bull before it is selected (at castration) is birth weight.

Estimates of variance and covariance components between traits and their respective DGV indicated that heritabilities of the DGV were greater than 0.80 but less than the expected value of 1, when DGV were obtained by K-means clustering and cross-validation (Table 8). The estimated heritabilities for DGV were higher (greater than 0.99) when DGV were obtained by random clustering and cross-validation (data not shown). Heritabilities less than 1 for the DGV obtained by K-means clustering and cross-validation show that the estimated marker effects were not consistent between training sets due to the differences in relatedness between the training and validation groups when five separate models were used to estimate the DGV of animals in each group. However, essentially the same extent of pedigree relatedness is expected when groups are constructed randomly (i.e., groups do not represent subpopulations) which leads to the heritability of DGV being close to 1. The estimated correlations between trait and respective DGV were higher than those reported by MacNeil et al. [15] for the same traits in Angus cattle, because they used a 384 SNP subset derived from the Illumina BovineSNP50 BeadChip to obtain DGV and validated in a single group (correlations of 0.68, 0.73, and 0.80 in comparison to 0.50, 0.65 and 0.54 for fat thickness, marbling and carcass weight, respectively, Table 8). Estimates of heritability for traits using the bivariate animal model were lower than the corresponding heritabilities reported by AAA or obtained by the weighted univariate animal model using DEBV (results not shown).

This could be due to the dependency between DEBV and DGV when the DGV of animals in one group were predicted from the DEBV of animals in the four other groups. Although five separate models were used to predict DGV, the DGV of individuals in one group are linear combination of the DEBV of individuals in the other groups which makes the covariance matrix between DEBV and DGV close to singular in the bivariate animal model analysis. More studies are needed to overcome this problem.

We used 5-fold cross-validation to evaluate the accuracy of DGV. The advantage of multi-fold cross-validation is that it can retain large training and validation sets. However, in contrast to most previous studies, we used K-means clustering to minimize the genetic relationships between groups. The distribution of a_max _(maximum additive-genetic relationship) for individuals within each group indicated that a_max _has a high density around 0.5 (sire-son relationships) and 0.25 (half-sib relationships) but a low density between groups. The distribution of inbreeding coefficients within each group revealed that the Wye population and its descendants (group 5) was distinct from the other groups, with an average inbreeding coefficient of about 0.10 due to the closing of the herd 10 generations ago and this group had low average relationships to the other groups. Accuracies of DGV were generally lower for this group, although it had a larger training set size.

When validation was performed on the younger animals or in groups obtained by random clustering, the accuracies of DGV were much higher than when cross-validation was performed in the K-means defined groups because of the higher genetic relationships between the training and validation set individuals. The lower accuracy of DGV for maternal calving ease in the younger animals is likely the result of low accuracies of EBV (and DEBV) in the younger animals, as these young bulls have few if any daughters of sufficient age to produce calving ease information. The higher accuracy of DGV with random clustering over validation on younger animals is caused by the higher genetic relationships between the training and validation sets within the randomly formed groups. These results demonstrate that validation is sensitive to the choice of the validation sample and to the pedigree relationships between the animals contributing to the validation and training sets, and the accuracies of DGV are dependent on the strength of genetic relationships between the training and validation sets. Thus, on the one hand, a dynamic training population will maintain an approximately constant average genetic relationship between animals in the training set and younger animals available for selection, leading to the largest possible DGV accuracies. On the other hand, future selection candidates, which do not have close relatives in the training set, will have DGV with reduced accuracies. However, we anticipate that there will be greater LD between markers and QTL and thus less dependency of the accuracies of DGV on the genetic relationships between training and validation sets when the recently released Illumina BovineHD and Affymetrix BOS 1 panels are employed for genomic selection.

## Conclusion

This study applied genomic prediction to US Angus beef cattle. By minimizing the relationships between training and validation groups using K-means clustering, the accuracy of DGV ranged from 0.22 to 0.69, with an average 0.44 across 16 economically important traits. Accuracies ranged from 0.38 to 0.85 with an average of 0.65 when training and validation sets were created by random allocation. Estimates of genetic correlations between traits and their respective DGV (obtained by K-means clustering) ranged from 0.15 to 0.80. These results demonstrate the feasibility of developing DGV for Angus beef cattle and show that the accuracy of predictions will deteriorate as the relationship between animals in the training set and selection candidates decreases. This suggests that, when using the BovineSNP50 BeadChip in the American Angus beef cattle population, a dynamic training set will be required to maximize the accuracy of selection in young animals and that the accuracy of DGV for animals in a population will be improved by including their sires in the training set.

## Competing interests

SB and BW are employed by the Igenity Livestock Business Unit that markets DNA diagnostic tests to cattle breeders. This includes a product for genomic-enhanced EPD for Angus cattle derived from the analysis of a subset of animals used in this study. SLN is employed by The American Angus Association, a corporation organized as a non-profit institution that provides members with EPD based on pedigree, performance and sometimes genomic information. The other authors declare that they have no competing interests.

## Authors' contributions

MS performed the statistical analyses, applied the K-means method for clustering genotyped animals and wrote the first draft of the manuscript. DJG and JFT designed the experiment, supervised the study and critically contributed to the final version of manuscript. RDS, JCMD and RLF participated in discussion and reviewed the manuscript. The other authors contributed materials. All authors read and approved the final manuscript.
